# Bruchpilot and Synaptotagmin collaborate to drive rapid glutamate release and active zone differentiation

**DOI:** 10.3389/fncel.2015.00029

**Published:** 2015-02-05

**Authors:** Mila M. Paul, Martin Pauli, Nadine Ehmann, Stefan Hallermann, Markus Sauer, Robert J. Kittel, Manfred Heckmann

**Affiliations:** ^1^Department of Neurophysiology, Institute of Physiology, Julius-Maximilians-University WürzburgWürzburg, Germany; ^2^Carl-Ludwig-Institute for Physiology, University of LeipzigLeipzig, Germany; ^3^Department of Biotechnology and Biophysics, Julius-Maximilians-University WürzburgWürzburg, Germany

**Keywords:** Bruchpilot, active zone, neurotransmitter release, synaptic delay, presynaptic differentiation, synaptotagmin, *d*STORM

## Abstract

The active zone (AZ) protein Bruchpilot (Brp) is essential for rapid glutamate release at *Drosophila melanogaster* neuromuscular junctions (NMJs). Quantal time course and measurements of action potential-waveform suggest that presynaptic fusion mechanisms are altered in *brp* null mutants (*brp^69^*). This could account for their increased evoked excitatory postsynaptic current (EPSC) delay and rise time (by about 1 ms). To test the mechanism of release protraction at *brp^69^* AZs, we performed knock-down of Synaptotagmin-1 (Syt) via RNAi (*syt^KD^*) in wildtype (wt), *brp^69^* and *rab3* null mutants (*rab3^rup^*), where Brp is concentrated at a small number of AZs. At wt and *rab3^rup^* synapses, *syt^KD^* lowered EPSC amplitude while increasing rise time and delay, consistent with the role of Syt as a release sensor. In contrast, *syt^KD^* did not alter EPSC amplitude at *brp^69^* synapses, but shortened delay and rise time. In fact, following *syt^KD^*, these kinetic properties were strikingly similar in wt and *brp^69^*, which supports the notion that Syt protracts release at *brp^69^*synapses. To gain insight into this surprising role of Syt at *brp^69^* AZs, we analyzed the structural and functional differentiation of synaptic boutons at the NMJ. At ‘tonic’ type Ib motor neurons, distal boutons contain more AZs, more Brp proteins per AZ and show elevated and accelerated glutamate release compared to proximal boutons. The functional differentiation between proximal and distal boutons is Brp-dependent and reduced after *syt^KD^*. Notably, *syt^KD^* boutons are smaller, contain fewer Brp positive AZs and these are of similar number in proximal and distal boutons. In addition, super-resolution imaging via *d*STORM revealed that *syt^KD^* increases the number and alters the spatial distribution of Brp molecules at AZs, while the gradient of Brp proteins per AZ is diminished. In summary, these data demonstrate that normal structural and functional differentiation of *Drosophila* AZs requires concerted action of Brp and Syt.

## INTRODUCTION

Active zones (AZs) allow exquisite spatial and temporal control of vesicle fusion. Large multidomain proteins rich in coiled-coil sequences such as Bassoon, Piccolo and the CAST/ERC family member Brp are major structural and functional organizers of AZs (Südhof 2012). Their abundance appears to correlate positively with neurotransmitter release (Graf et al., 2009; Matz et al., 2010; Weyhersmüller et al., 2011; Ehmann et al., 2014; Peled et al., 2014).

At *Drosophila melanogaster* NMJs, Brp is crucial for synchronous glutamate release and the clustering of calcium channels at AZs (Kittel et al., 2006; Wagh et al., 2006). Linking the amount of Brp or Bassoon per AZ to the number and spatial arrangement of calcium channels may account for the correlation with release probability, e.g., in the context of synaptic homeostasis (Matz et al., 2010; Weyhersmüller et al., 2011; Ehmann et al., 2014). Slight increases in coupling distance in the 20–40 nm range reduce release probability dramatically while changing kinetic release parameters to a lesser extent (Neher, 1998; Eggermann et al., 2011; Schmidt et al., 2013; Vyleta and Jonas, 2014). Differences in coupling distance are therefore ideal for scaling the amount of release, whereas controlling its time course appears to require additional molecular mechanisms.

The main kinetic transmitter release parameters are synaptic delay, rise, and decay times. In the present study, we focus on synaptic delay and EPSC rise time. Notably, the latter is increased by more than 1 ms at synapses lacking Brp, while release probability drops by comparison only moderately (Kittel et al., 2006; Eggermann et al., 2011). This marked kinetic change appears disproportional to the reduction in release probability. While synaptic delay has not yet been analyzed at *brp^69^* synapses, it is usually fairly constant for a wide range of release probabilities (Barrett and Stevens, 1972; Datyner and Gage, 1980).

While the molecular mechanisms controlling release kinetics are complex and not well understood (Neher, 2010), it is clear that the vesicle protein Syt plays an important role (Brose et al., 2002; Young and Neher, 2009). As initially suggested more than 20 years ago (DiAntonio et al., 1993; Littleton et al., 1993; Geppert et al., 1994), Syt is crucial for triggering release and may act both as a calcium sensor and a vesicle fusion clamp. In fact, its role may change from clamp to sensor upon calcium influx into the presynaptic terminal (DeBello et al., 1993; Walter et al., 2011).

To clarify the molecular mechanisms that shape the time course of release we analyzed the interaction between Brp and Syt. We find that in addition to prolonged EPSC rise time, synaptic delay is strongly increased at *brp^69^* synapses. Interestingly, whereas Syt is necessary for the increase in both kinetic parameters, it has little effect on the amount of transmitter released from *brp^69^* AZs. Following up on the functional interaction of Brp and Syt, our data suggest central roles of these two proteins in the spatial differentiation of AZs and reveal that the number of AZs per bouton, as well as the number and distribution of Brp molecules per AZ is Syt-dependent.

## MATERIALS AND METHODS

### FLY STOCKS

**FIGURE 1:**
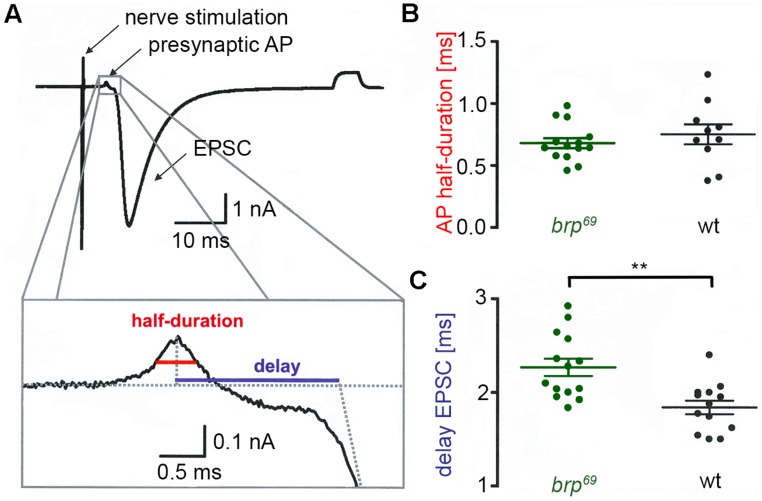
**Presynaptic action potential and synaptic delay in *brp^**69**^*. (A)** Focal recording from a *Drosophila* wt NMJ. Nerve stimulation (left arrow) elicited an action potential in the suction electrode which traveled to the presynaptic bouton under the focal electrode and lead to deflections (presynaptic AP, second arrow) prior to the large compound EPSC (third arrow). A subthreshold pulse through the focal electrode elicited the final upward deflection (right side of the trace and allowed to measure electrode and seal resistance). The lower panel shows the enlarged presynaptic AP and illustrates how half-duration of the positive AP deflection (red) and synaptic delay (blue) were determined. **(B)** While AP half-duration is unchanged between *brp^69^* (green) and wt (black), **(C)** synaptic delay is prolonged in *brp^69^*. Shown are single values (dots) and mean ± SD.

*Drosophila* larvae were raised in vials on standard corn meal (Ashburner, 1989) at 25°C for focal recordings in **Figure 1** (wt and *brp^69^)* or at 29°C for reliable RNAi expression in all other experiments (mutant and control groups). For Syt RNAi, we expressed *syt1_RNAi^8875^* Vienna *Drosophila* Resource Center (VDRC) specifically in motor neurons (*ok6-GAL4/+; UAS-syt1_RNAi^8875^/+)* or panneuronally (*elav-GAL4/UAS-syt1_RNAi^8875^)* using the binary *UAS-GAL4* expression system (Brand and Perrimon, 1993). *brp^69^* and *rab3^rup^* were used as previously described (Kittel et al., 2006; Graf et al., 2009). To combine *brp^69^* and *rab3^rup^* with *syt1_RNAi^8875^* the following lines were generated: *brp^69^ok6-GAL4/ df(2R)BSC29; UAS-syt1_RNAi^8875^/+* and *brp^69^/df(2R)BSC29; elav-GAL4/UAS-syt1_RNAi^8875^ (brp^69^, syt^KD^); rab3^rup^/df(2R)ED2076 ok6-GAL4; UAS-syt1_RNAi^8875^/+* and* rab3^rup^/df(2R)ED2076; elav-GAL4/UAS-syt1_RNAi^8875^ (rab3^rup^, syt^KD^); ok6-GAL4/+* and *elav-GAL4/+* served as wt controls. For focal recordings, GFP was expressed in presynaptic terminals: *ok6-GAL4/+; UAS-CD8::GFP/+ (*wt*), brp^69^ok6-GAL4/df(2R)BSC29;UAS-CD8::GFP/+ (brp^69^) and ok6-GAL4/+; UAS-CD8::GFP/UAS-Syt1_RNAi^8875^ (syt^KD^).* For non-allelic non-complementation the following genotypes were used: *w^1118^* (wt), *syt1^AD4^/+* (DiAntonio et al., 1993), *brp^69^/+* (Kittel et al., 2006) and *syt1^AD4^/ brp^69^* (*syt^AD4^+/+brp^69^*).

### PHYSIOLOGICAL SOLUTION AND PREPARATION

The composition of the extracellular, physiological, hemolymph-like saline (HL-3, Stewart et al., 1994) was (in mM): NaCl 70, KCl 5, MgCl_2_ 20, NaHCO_3_ 10, trehalose 5, sucrose 115, HEPES 5, CaCl_2_ as indicated, pH adjusted to 7.2. Wandering male third instar larvae were dissected in HL-3 without CaCl_2_. All experiments were carried out at NMJs formed on ventral abdominal muscles 6/7 in segments A2 and A3.

### FOCAL RECORDINGS

Macropatch recordings in **Figure 1** were performed in HL-3 saline containing 1 mM [Ca^2+^]_Ex_ essentially as reported previously (Pawlu et al., 2004). Bath temperature was kept constant at 18 ± 0.5°C using a Peltier element (27 W, Conrad Electronic) glued to the bath inflow with heat-conductive paste (Fischer Elektronik). EPSCs were elicited using a 0.2 Hz nerve-stimulation protocol with 0.2 ms pulse duration and amplitudes slightly above the threshold for eliciting an action potential via a suction electrode (filled with extracellular solution). Recording electrodes with openings of about 5–10 μm diameter below the tip had resistances of 250 kΩ when filled with HL-3. About 20 EPSCs were recorded per site and analyzed with IgorPro 5.04 (Wavemetrics). The data were digitally filtered at 3 kHz (Gaussian filter), baseline subtracted and the average of all failures was subtracted from the currents. AP durations were measured at half amplitude of the positive deflection (Dudel, 1965) and synaptic delay was measured from the peak of the AP to the point at which the back extrapolation of the EPSC current rising phase crossed the baseline (**Figure 1**). Focal recordings in **Figure 6** were performed in HL-3 saline containing 0.5 mM [Ca^2+^]_Ex_. Bath temperature was kept constant at 20 ± 1°C. Focal electrodes (resistances 600 ± 50 kΩ when filled with HL-3 solution) were positioned on proximal or distal type Ib boutons of muscles 6/7. EPSCs were elicited using a 0.2 Hz nerve-stimulation protocol with 0.2 ms pulse duration and 7 V amplitude. Traces were low-pass filtered at 20 kHz, and recorded and stored with Patchmaster using an EPC10 double patch clamp amplifier (HEKA electronics). About 60 EPSCs were averaged per site and analyzed with Igor Pro 6.05 (Wavemetrics). 10 mg EGTA-AM (membrane permeable tetraacetoxymethyl ester of ehtyleneglycol-bis(ß-aminoethyl)-N,N,N’,N’-tetraacetic acid, Calbiochem Germany) was dissolved in DMSO with 20% Pluronic (Invitrogen) to obtain a stock solution of 10 mM EGTA. This stock solution was diluted 1:100 with calcium-free HL-3 and applied to the dissected preparation for 10 min. After incubation preparations were washed for 5 minutes with HL-3 (Müller et al., 2012) and recordings were performed in HL-3 containing 1.0 mM [Ca^2+^]_Ex_ as described above.

### TWO-ELECTRODE VOLTAGE CLAMP RECORDINGS (TEVC)

Two-electrode voltage clamp-recordings (**Figure 3**) were performed essentially as previously described (Kittel et al., 2006) using an Axo Clamp 2B amplifier (Axon Instruments, Molecular Devices). All measurements were made from muscle 6 at 21 ± 1°C bath temperature. Intracellular electrodes were filled with 3 M KCl and had resistances of 12–15 MΩ. V_holding_ was -60 mV for evoked EPSCs. Only cells with an initial membrane potential of at least -50 mV and ≥4 MΩ input resistance were analyzed. Synaptic responses were generated by pulses of 0.3 ms length and 5–10 V amplitude, applied via a suction electrode (filled with extracellular solution) and low-pass filtered at 10 kHz. We applied a 0.2 Hz stimulation protocol, averaged 20 EPSCs per muscle cell and analyzed the data with Clampfit (Axon Instruments, Molecular Devices).

### IMAGING

Larvae were dissected in ice-cold HL-3 standard saline without CaCl_2_, fixed with 4% paraformaldehyde in 0.1 M phosphate buffered saline (PBS) for 10 min and blocked with PBT (PBS containing 0.05% Triton X-100, Sigma) including 5% natural goat serum (Dianova) for 30 min. Primary antibodies were added for overnight staining at 4°C. After three washing steps with PBS (20 min each), preparations were incubated with secondary antibodies for 2–4 h at room temperature followed by three washing steps with PBS. Filets were mounted using Vectashield (Vector Laboratories) and images were aquired using an Apotome System (Zeiss, Axiovert 200M Zeiss, objective 63x, NA 1.4, oil). Antibodies were used at the following concentrations: mouse monoclonal antibody (mAb) Brp^Nc82^ (1:250), Alexa Fluor 488-conjugated goat α-mouse (Invitrogen) and Cy3-conjugated goat α-horseradish peroxidase (HRP, Jackson Immuno Research) antibodies (1:250), rabbit α-Dsyt-CL1 (Mackler et al., 2002) and Cy3-conjugated goat α-rabbit (Jackson Immuno Research, 1:250) antibodies. Z-stacks of 15–20 single images taken every 250 nm were maximum projected and analyzed in ImageJ (1.440, NIH). Brp puncta per NMJ and per bouton were quantified manually. Using the three terminal boutons of type Ib and Is branches the respective structural gradient was analyzed. Distal boutons were located at the end of bouton chains, while proximal boutons were closer to the entry site of the motor neuron. Bouton area, length (along chain axis) and width (90° to length) were measured using α-HRP stainings.

### dSTORM (*DIRECT* STOCHASTIC OPTICAL RECONSTRUCTION MICROSCOPY)

**FIGURE 2:**
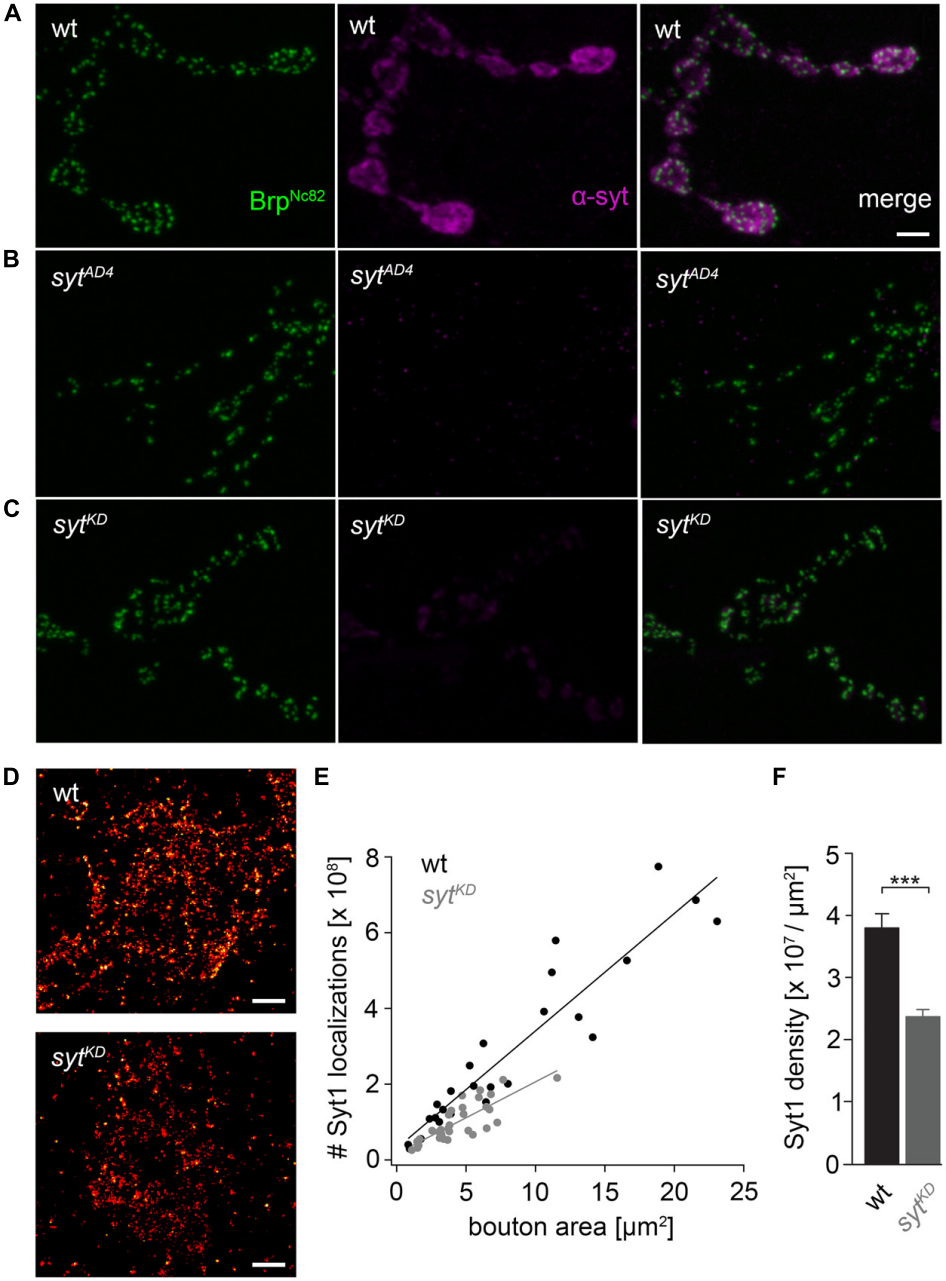
**Ribonucleic acid interference decreases presynaptic Syt protein levels. (A)** *Drosophila* wt NMJ on larval abdominal muscles 6/7 stained with antibodies against Brp (Brp^Nc82^, green) and Synaptotagmin (α-Syt, magenta). Brp demarks individual AZs, whereas Syt labels synaptic vesicles predominately distributed around the bouton circumference. **(B)** The α-Syt signal is absent in Syt null mutans (*syt^AD4^*) and **(C)** strongly reduced after Syt knock-down (*syt^KD^*) through presynaptically (*ok6-GAL4*) driven RNA interference (*UAS-syt1-RNAi^8875^*). **(D)** *d*STORM images of single presynaptic boutons stained against Syt1. **(E)** Number of single-fluorophore localization events by *d*STORM. Syt1 localizations plotted against bouton area for wt (black) and *syt^KD^* (gray) with respective regression lines. Dots represent values for single boutons. **(F)** Summary bar graph indicates the decrease of Syt1 density in boutons of similar size (below 10 μm^2^) to 62.38% in *syt^KD^* compared to wt (*p* < 0.001). Scale bar = 2 μm **(A–C)** and 300 nm **(D)**.

Larvae were dissected, fixed and washed as described above and super-resolution imaging was performed essentially as previously reported (Ehmann et al., 2014). Preparations were incubated with mAb Brp^Nc82^ (1:2000) and secondary antibody goat α-mouse F(ab’)_2_ fragments (A10534, Invitrogen) labeled with Cy5-NHS (PA15101, GE Healthcare) at a concentration of 5.2 × 10^-8^ M or with rabbit α-Dsyt-CL1 (1:10000, Mackler et al., 2002) and secondary antibody goat α-rabbit F(ab’)_2_ fragments labeled with Alexa Fluor 647 (1:500, Invitrogen). Boutons were visualized with Alexa Fluor 488 conjugated goat α-horseradish-peroxidase antibody (α-HRP, 1:250, Jackson Immuno Research). After staining, larval preparations were incubated in 100 mM Mercaptoethylamin (MEA, Sigma-Aldrich) buffer in PBS, pH 7.8–7.9 to allow reversible switching of single fluorophores during data acquisiton (van de Linde et al., 2008). Images were acquired using an inverted microscope (Olympus IX-71, 60x, NA 1.45, oil immersion) equiped with a nosepiece-stage (IX2-NPS, Olympus). 644 nm (iBEAM-SMART-640-S, Toptica), and 488 nm (iBEAM-SMART-488-S, Toptica) lasers were used for excitation of Cy5 and Alexa Fluor488, respectively. Laser beams were passed through a clean-up filter (Brightline HC 642/10, Semrock, and ZET 488/10, Chroma, respectively) and two dichroic mirrors (Laser-MUX BS 514-543 and HC-quadband BP, Semrock) onto the probe. The emmitted fluorescence was filtered with a quadband-filter (HC-quadband 446/523/600/677, Semrock) and divided onto two cameras (iXon Ultra DU-897-U, Andor) using a dichroic mirror (HC-BS 640 imaging, Semrock). In addition, fluorescence was filtered using a longpass- (Edge Basic 635, Semrock) or bandpass-filter (Brightline HC 525/50, Semrock) for red and green channels, respectively. Pixel sizes were 126 nm (red) and 128 nm (green). Single fluorophores were localized and high resolution-images were reconstructed with rapi*d*STORM (Heilemann et al., 2008; Wolter et al., 2010; van de Linde et al., 2011; Wolter et al., 2012; www.super-resolution.de). Only fluorescence spots with more than 1000 photons were analyzed (10 nm /pixel sub-pixel binning). Data were processed with ImageJ (1.440, NIH), AZ area and Brp localizations per AZ were analyzed as previously described Ehmann et al. (2014). AZs were assigned to single boutons using the α-HRP signal. For Syt1 quantification (**Figure 2**) we determined localization counts in single type Ib boutons that were defined according to the α-HRP signal. Localization densities were analyzed only in boutons with areas <10 μm.

### STATISTICAL ANALYSIS

Statistical analyses were performed with Sigma Plot 12 (Systat Software) using the non-parametric Mann–Whitney rank sum test. Linear fits for mean ± SEM Brp localizations per AZ (**Figure 9**) were made in Igor Pro (Wavemetrics) and statistical analysis was performed using the non-parametric Spearman correlation coefficient. Asterisks indicate the significance level (^∗^*p* < 0.05, ^∗∗^*p* < 0.01, ^∗∗∗^*p* < 0.001). Data are reported as mean ± SEM unless indicated otherwise and *n* denotes sample number.

## RESULTS

### SYNAPTIC DELAY IS INCREASED IN *brp^**69**^*

We performed focal recordings using macropatch electrodes which allow simultaneous measurements of presynaptic action potentials (AP) and synaptic release (Dudel, 1965). EPSCs evoked by 0.2 Hz nerve stimulation were recorded at *brp^69^* and wt larval NMJs on muscles 6/7 to measure half-duration of the positive AP deflection and synaptic delay (**Figure 1A**). Whereas AP wave form was unchanged in *brp^69^* compared to wt, synaptic delay was significantly increased (2.27 ± 0.3 ms and 1.8 ± 0.3 ms mean ± SD, *p* = 0.0014, *n* = 14, and 13 for *brp^69^* and wt, **Figures 1B,C**). As quantal time course is normal in *brp^69^* (Kittel et al., 2006), the increase in release kinetics at *brp^69^* synapses is likely due to alterations in presynaptic fusion mechanisms.

### IMPACT OF SYT ON DIFFERENT AZ STATES

**FIGURE 3:**
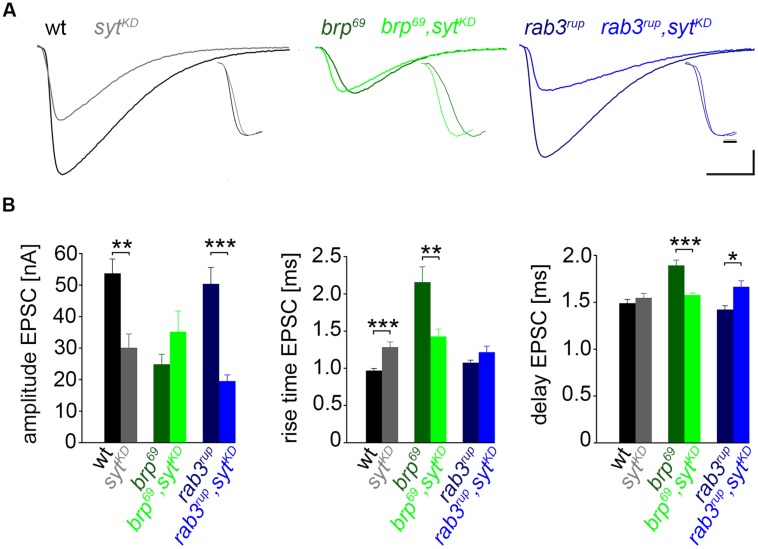
**Effect of *syt^**KD**^* on amplitude and kinetics of the postsynaptic response. (A)** Two-electrode voltage clamp recordings in physiological solution with 1.0 mM [Ca^2+^]_Ex_ and 0.2 Hz nerve stimulation. Traces from wt (black), *brp^69^* (dark green), and *rab3^rup^* (dark blue) are shown superimposed with recordings of the respective genotypes after *syt^KD^* (brighter shades). Insets show normalized events to highlight the differences in synaptic delay and postsynaptic current rise time. Scale bars = 5 ms, 10 nA (main traces), and 1 ms (insets). **(B)** Summary bar graphs for mean ± SEM amplitude, current rise time and synaptic delay for all six genotypes, color coded as in **(A)**.

We used wt, *brp^69^* and *rab3^rup^* to define explicit AZ conditions: (i) normal organization, (ii) disorganized lacking Brp (Kittel et al., 2006), and (iii) large accumulation of Brp proteins (Graf et al., 2009; Ehmann et al., 2014), respectively. To determine the impact of the putative calcium sensor Syt on synchronous transmitter release in the context of different AZ states we combined these genotypes with *syt^KD^*. Protein levels of endogeneous Syt were decreased via RNAi (*syt1-RNAi^8875^*, see experimental procedures). By engaging the binary *UAS-Gal4* expression system (Brand and Perrimon, 1993), *syt1-RNAi* was driven in larval glutamatergic motor neurons or panneuronally. To confirm that presynaptic Syt expression was reduced by this strategy, immunostainings of larval NMJs were performed using an antiserum against Syt1 (Mackler et al., 2002; **Figure 2**). Whereas presynaptic terminals of *syt^AD4^* were completely devoid of Syt, there was residual though heavily reduced protein expression in *syt^KD^* compared to wt (**Figure 2C**). Furthermore, we quantified the protein reduction following Syt knock-down with *d*STORM (Ehmann et al., 2014). Comparison of Syt1 localization numbers in boutons of similar size in wt and *syt^KD^* revealed a reduction to 62.38% in *syt^KD^* (**Figures 2D–F**). To address the functional consequences of *syt^KD^* at wt, *brp^69^*, and *rab3^rup^* synapses, postsynaptic currents in response to low-frequency nerve stimulation were recorded in two-electrode voltage clamp mode (TEVC) from larval ventral abdominal muscles 6/7 (**Figure 3**). Both panneuronal (**Figure 3**) and motoneuronal (data not shown) RNAi expression gave essentially comparable results. At wt synapses, *syt^KD^* decreased EPSC amplitude and lengthened rise time (amplitude: 30.0 ± 4.5 nA and 53.6 ± 4.7 nA, *p* = 0.002; rt: 1.3 ± 0.1 ms and 1.0 ± 0.03 ms, *p* < 0.001; *n* = 11 and 17 for *syt^KD^* and wt, **Figure 3B**) consistent with the role of Syt as a sensor for fast release (DiAntonio et al., 1993; Littleton et al., 1993). Similarly, at *rab3^rup^* synapses, *syt^KD^* reduced the amplitude and increased the delay of postsynaptic responses (amplitude: 19.5 ± 2.1 nA and 50.3 ± 5.3 nA, *p* < 0.001; delay: 1.7 ± 0.1 ms and 1.4 ± 0.04 ms, *p* = 0.013; *n* = 14 and 10 for *rab3^rup^*, *syt^KD^*, and *rab3^rup^*). Strikingly, *syt^KD^* at *brp^69^* synapses left current amplitudes unchanged (35.1 ± 6.7 nA and 24.8 ± 3.3 nA, *p* > 0.05) and in fact accelerated EPSC rise time and delay (rt: 1.4 ± 0.1 ms and 2.2 ± 0.2 ms, *p* = 0.008; delay: 1.6 ± 0.03 ms and 1.9 ± 0.1 ms, *p* < 0.001; *n* = 12 and 11 for *brp^69^*, *syt^KD^*, and *brp^69^*). These results illustrate that Syt is necessary for efficient and rapid vesicle fusion at AZs with normal or increased Brp levels. In contrast, vesicle release from* brp^69^* AZs appears less dependent on Syt. We did not find any changes in size or kinetics of quantal events in *brp^69^* and *brp^69^*, *syt^KD^* that could explain these effects (amplitude: 0.89 ± 0.04 nA and 0.90 ± 0.04 nA; rt: 1.0 ± 0.06 ms and 1.0 ± 0.04 ms; tau: 6.02 ± 0.6 ms and 7.2 ± 0.4 ms; all *p* > 0.05; *n* = 10 and 14 for *brp^69^* and *brp^69^*, *syt^KD^*, respectively). Thus, the changes in release kinetics following *syt^KD^* suggest that Syt protracts release at *brp^69^* AZs.

### REDUCED EGTA SENSITIVITY IN *brp^*69*^, syt^KD^*

**FIGURE 4:**
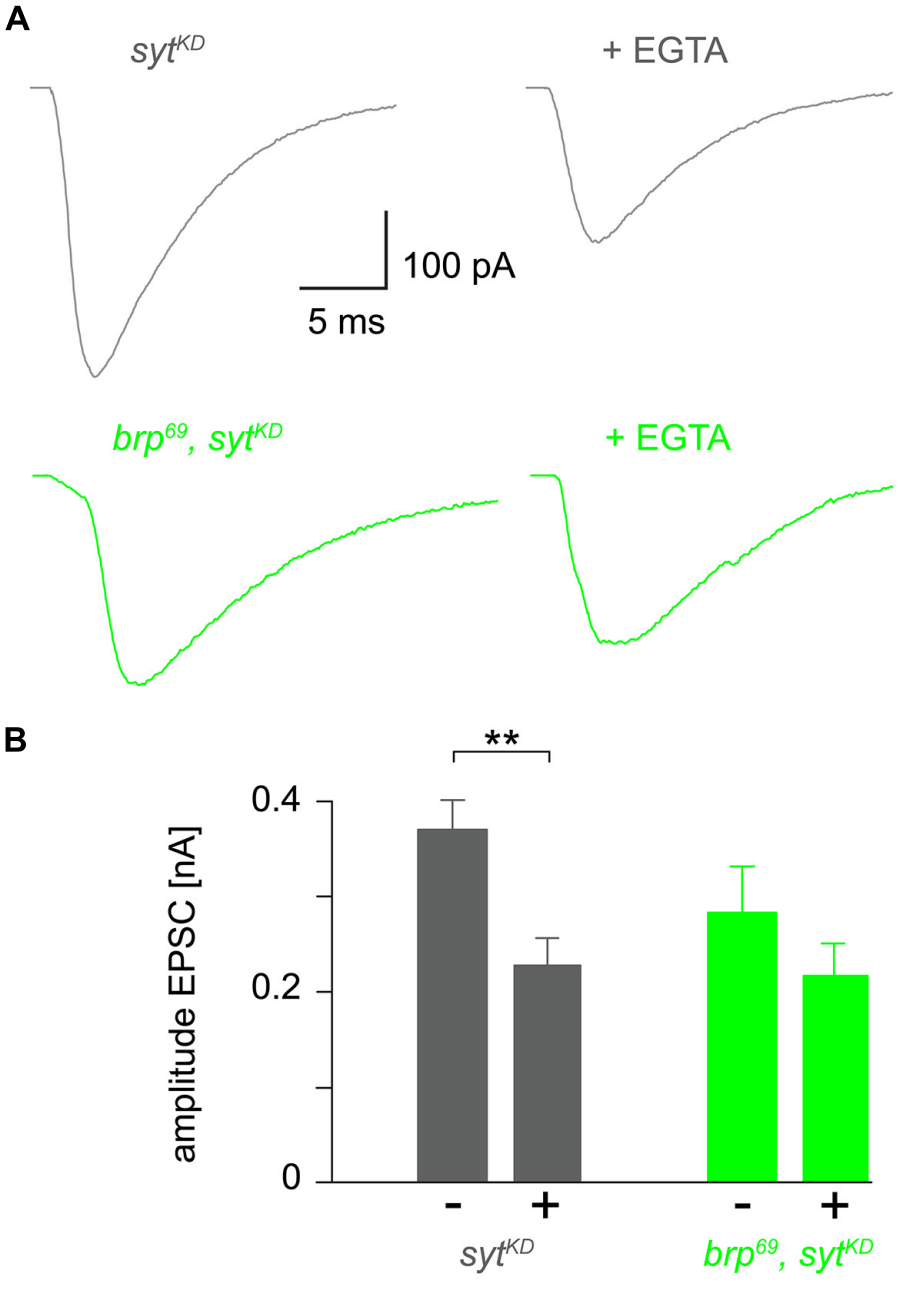
**Reduced EGTA sensitivity in *brp^**69**^, syt^**KD**^*. (A)** Focally recorded, averaged EPSCs from *syt^KD^* (gray) and *brp^69^, syt^KD^* (light green) NMJs under control conditions and after incubation with 100 μM EGTA-AM for 10 min (+EGTA). **(B)** Summary bar graph of EPSC amplitudes without (–) or with (+) EGTA-AM in both genotypes.

To further clarify how *syt^KD^* affects release we tested the influence of EGTA-AM in *syt^KD^* and *brp^69^*, *syt^KD^* in focal recordings (**Figure 4**, Kittel et al., 2006). Consistent with earlier work (Maximov and Südhof, 2005) release in *syt^KD^* was significantly reduced (0.37 ± 0.03 nA and 0.22 ± 0.03 nA, *p* = 0.002, *n* = 16 each), whereas the reduction was not significant in *brp^69^*, *syt^KD^* (0.28 ± 0.05 nA and 0.21 ± 0.03 nA, *p* > 0.05, *n* = 17, and 10 without and with EGTA). This is in contrast to the findings described in Kittel et al. (2006) for *brp^69^* and suggests that Syt knock-down reduces coupling distance in *brp^69^* and that Syt’s role in positional priming (Young and Neher, 2009) requires Brp.

### BRP IS DISTRIBUTED UNEVENLY AT WT AND *rab3^rup^* NMJs

**FIGURE 5:**
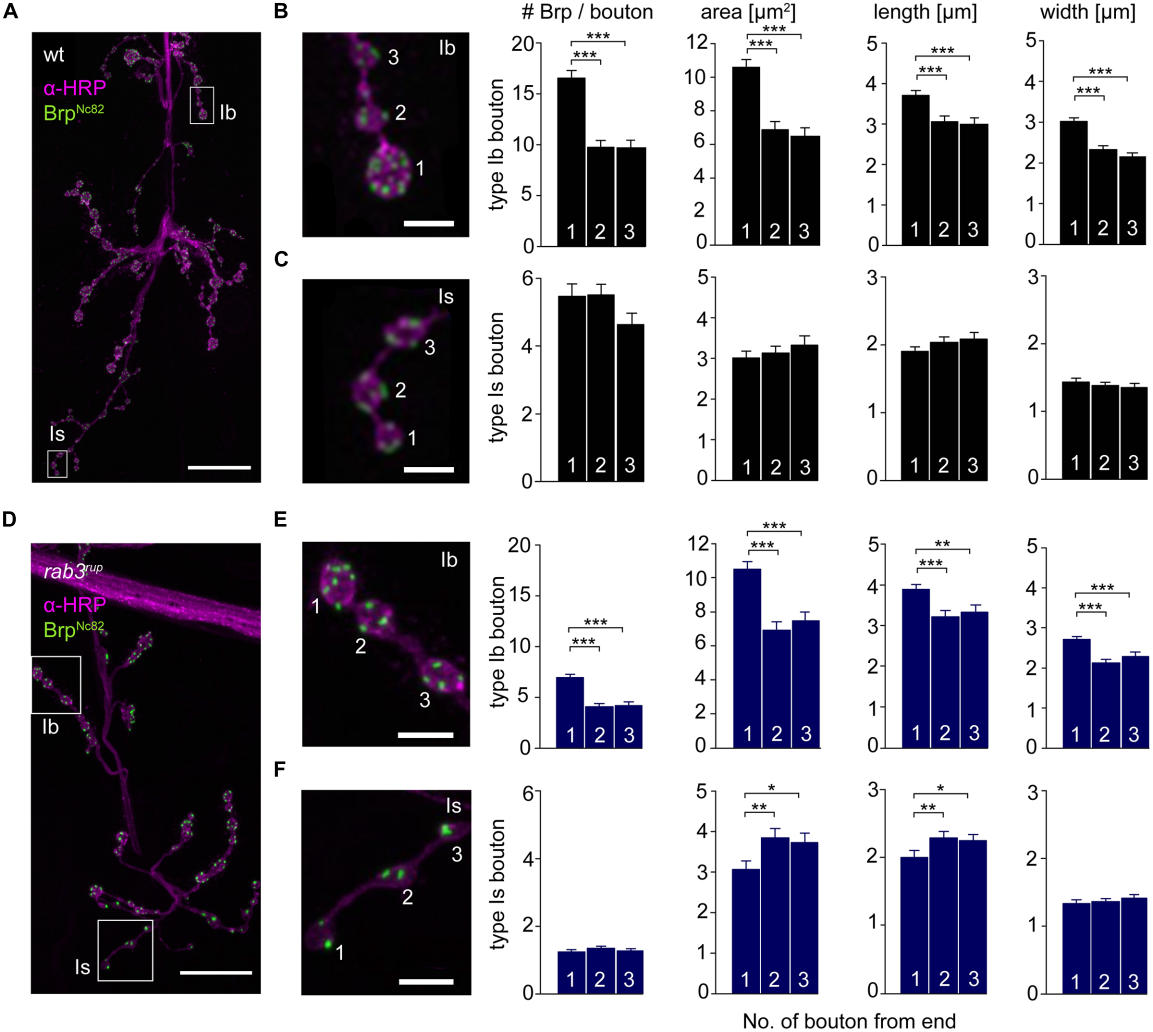
**Type Ib and type Is bouton morphometry in wt and *rab3^**rup**^*. (A)** *Drosophila* wt NMJ stained with α-HRP against neuronal membranes (magenta) and mAb Brp^Nc82^ against Brp (green). White boxes indicate terminal three boutons of type Ib and type Is branches. **(B,C)** Magnification of boxed regions in **(A)**. Summary bar graphs for the number of Brp puncta per bouton and bouton area, length and width of the terminal three boutons. **(D)** *Drosophila rab3^rup^* NMJ as in **(A)**. Neurite passing by the NMJ at the top. **(E,F)** Magnification of boxed regions in **(D)** show the terminal three boutons of respective type Ib and type Is endings. Summary bar graphs for the number of Brp puncta per bouton and area, length and width of the terminal three boutons. Scale bars = 20 μm **(A,D)** and 5 μm **(B,C,E,F)**.

The innervation of ventral abdominal muscles 6/7 is shared between two functionally distinct motoneurons in *Drosophila* larvae (Karunanithi et al., 2002): the MN6/7b-Ib (RP3) neuron gives rise to large type Ib boutons (Atwood et al., 1993), whereas the MNSNb/d-Is neuron forms smaller type Is boutons (Kurdyak et al., 1994; Lnenicka and Keshishian, 2000; Hoang and Chiba, 2001). We performed immunostainings and counted the number of Brp puncta per bouton as an estimate of the number of AZs. In addition, staining against horseradish-peroxidase (HRP) was used to measure dimensions of presynaptic arborizations (Jan and Jan, 1982; **Figure 5**). We found an uneven Brp distribution in wt type Ib motoneurons with more Brp positive AZs in distal than in proximal boutons (16.5 ± 0.8, 9.8 ± 0.7, 9.6 ± 0.8, *p* < 0.001, *n* = 115 (1), 70 (2), 70 (3), **Figure 5B**). In addition, distal type Ib boutons were largest along the bouton chain (10.6 ± 0.5 μm^2^, 6.9 ± 0.5 μm^2^, 6.5 ± 0.5 μm^2^, *p* < 0.001). Interestingly, this gradient was not found in type Is boutons regarding both AZ number [5.5 ± 0.4, 5.5 ± 0.3, 4.6 ± 0.3, *p* > 0.05, *n* = 73 (1), 73 (2), 73 (3)] and bouton area (3.0 ± 0.2 μm^2^, 3.1 ± 0.2 μm^2^, 3.3 ± 0.2 μm^2^, *p* > 0.05, **Figure 5C**). Our data are in line with previous work analyzing functional (Guerrero et al., 2005; Peled and Isacoff, 2011) and structural properties (Ehmann et al., 2014) of the NMJ. Furthermore, the number of AZs per bouton was decreased at *rab3^rup^* NMJs compared to wt (**Figures 5D–F**). This matches earlier work, showing that Rab3 controls the distribution of Brp at the NMJ with decreased AZ numbers per NMJ and increased Brp levels per AZ in *rab3^rup^* (Graf et al., 2009). However, the structural gradient regarding AZ number per bouton and bouton size was still present in *rab3^rup^* type Ib axons [Brp: 7.0 ± 0.3, 4.1 ± 0.3, 4.2 ± 0.3; area: 10.5 ± 0.5 μm^2^, 6.9 ± 0.5 μm^2^, 7.5 ± 0.5 μm^2^, *p* < 0.001, respectively, *n* = 117 (1), 57 (2), 57 (3), **Figure 5E**]. Thus, a structural gradient is present along the MN6/7b-Ib motor neuron with larger distal than proximal type Ib boutons. In addition, Brp is unevenly distributed in type Ib boutons of wt and *rab3^rup^* NMJs.

### SYT AND BRP ARE ESSENTIAL FOR FUNCTIONAL PRESYNAPTIC DIFFERENTIATION

**FIGURE 6:**
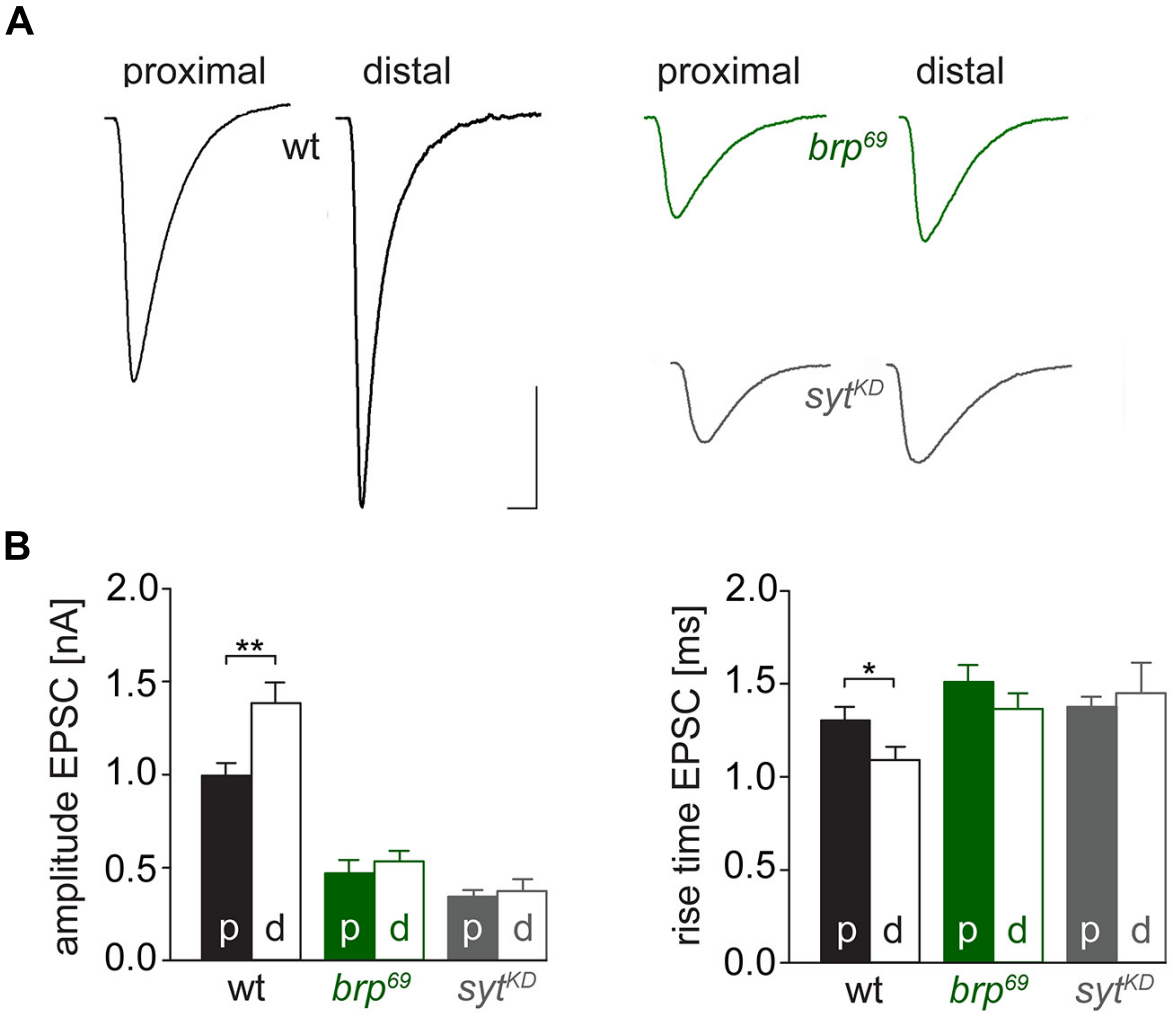
**Functional differentiation of proximal and distal type Ib boutons. (A)** Averaged EPSCs elicited by 0.2 Hz nerve stimulation in physiological solution containing 0.5 mM [Ca^2+^]_Ex_ and recorded focally from proximal and distal type Ib boutons in wt (black), *brp^69^* (green) and *syt^KD^* (gray). Scale bars = 0.5 ms, 1 nA. **(B)** Summary bar graphs for EPSC amplitudes and current rise times (mean ± SEM) for the three genotypes with p, proximal and d, distal, color coded like in **(A)**.

We used focal electrodes as these can be selectively placed on a subset of presynaptic boutons to improve spatial resolution of synaptic measurements. Boutons were visualized by GFP-expression (Pawlu et al., 2004) and postsynaptic currents of proximal and distal type Ib boutons were measured in response to low-frequency nerve stimulation in 0.5 mM [Ca^2+^]_Ex_ (**Figure 6**). Distal boutons of wt NMJs showed larger EPSC amplitudes and shorter rise times than proximal boutons (amplitude: 1.4 ± 0.1 nA and 1.0 ± 0.1 nA, *p* = 0.005; rt: 1.1 ± 0.07 ms and 1.3 ± 0.07 ms, *p* = 0.032, *n* = 24, and 33, **Figure 6B**). In contrast, at both *brp^69^* and *syt^KD^* NMJs amplitude and kinetics of postsynaptic currents were comparable in distal and proximal boutons (*n* = 10 and 11 for *brp^69^* and 11 and 13 for *syt^KD^*, **Figure 6B**). These results reveal that Brp and Syt are both essential for the functional differentiation of the NMJ.

### SYT AND BRP INTERACT GENETICALLY

**FIGURE 7:**
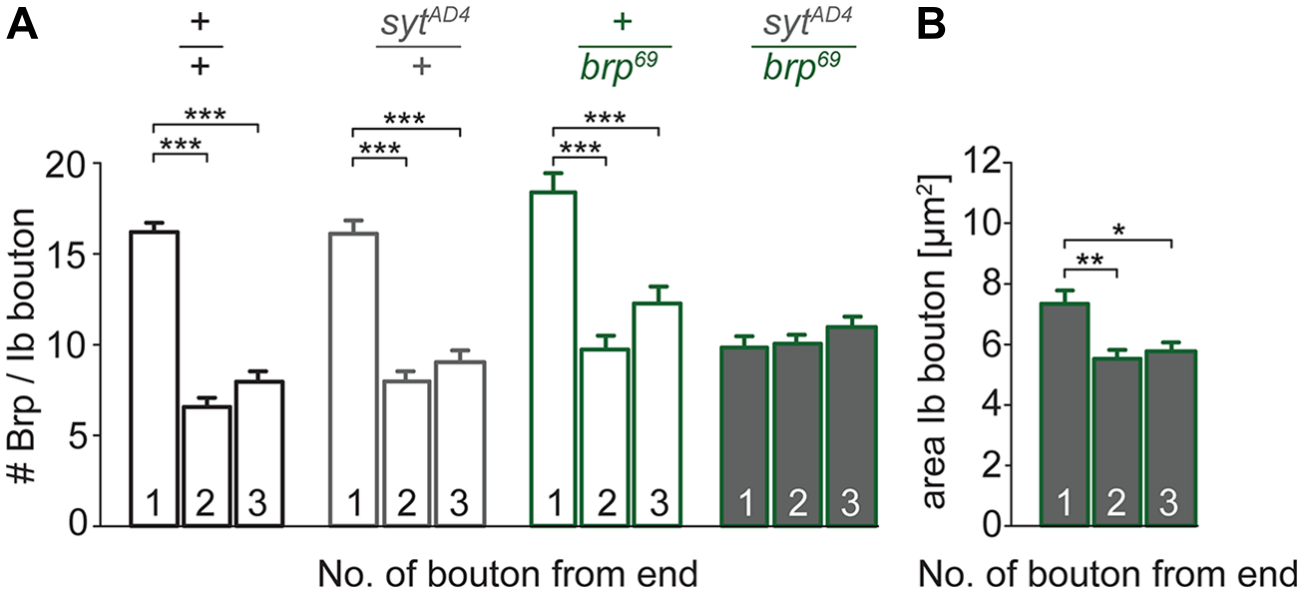
**Genetic interaction between *brp* and *syt*. (A)** Summary bar graphs for the number of Brp puncta per terminal three type Ib boutons of wt (*+/+,* black), heterozygous *syt^AD4^* (*syt^AD4^/+*, gray), heterozygous *brp^69^* (+/*brp^69^*, green) and trans-heterozygous *syt^AD4^/brp^69^* larvae (gray with green edge). **(B)** Summary bar graph for the area of the three terminal type Ib boutons in *syt^AD4^/brp^69^* trans-heterozygotes.

To test for a genetic interaction between Brp and Syt we analyzed non-allelic non-complementation (Yook et al., 2001). This genetic strategy tests for the ability of two recessive mutations to complement one another for a specific phenotype. We studied heterozygous animals carrying either one copy of the *syt* null allele *Syt^AD4^* (DiAntonio et al., 1993) or the *brp* null allele *brp^69^* (Kittel et al., 2006) and trans-heterozygous animals carrying both. We analyzed the structural gradient along the MN6/7b-Ib motor axon regarding AZ number and bouton dimensions (**Figure 7**). Whereas in heterozygous animals distal boutons contained more Brp puncta than proximal boutons (*p* < 0.001, *n* = 95 (1), 72 (2), 72 (3) for *syt^AD4^*; *p* < 0.001, *n* = 51 (1), 40 (2), 40 (3) for *brp^69^*), this gradient was lost in trans-heterozygous animals (9.8 ± 0.5, 10.0 ± 0.4, 11.0 ± 0.5, *p* > 0.05, *n* = 81 (1), 81 (2), 81 (3), **Figure 7A**). Interestingly, distal boutons of trans-heterozygous animals were still largest along the bouton chain (7.3 ± 0.4 μm^2^, 5.5 ± 0.3 μm^2^, 5.8 ± 0.3 μm^2^, *p* = 0.003 and 0.014 for (2) and (3), **Figure 7B**). We conclude that regarding AZ distribution both mutations fail to complement one another, suggesting a direct interaction of Brp and Syt or an action of the two proteins in the same functional pathway.

### SYT GUIDES AZ DISTRIBUTION

**FIGURE 8:**
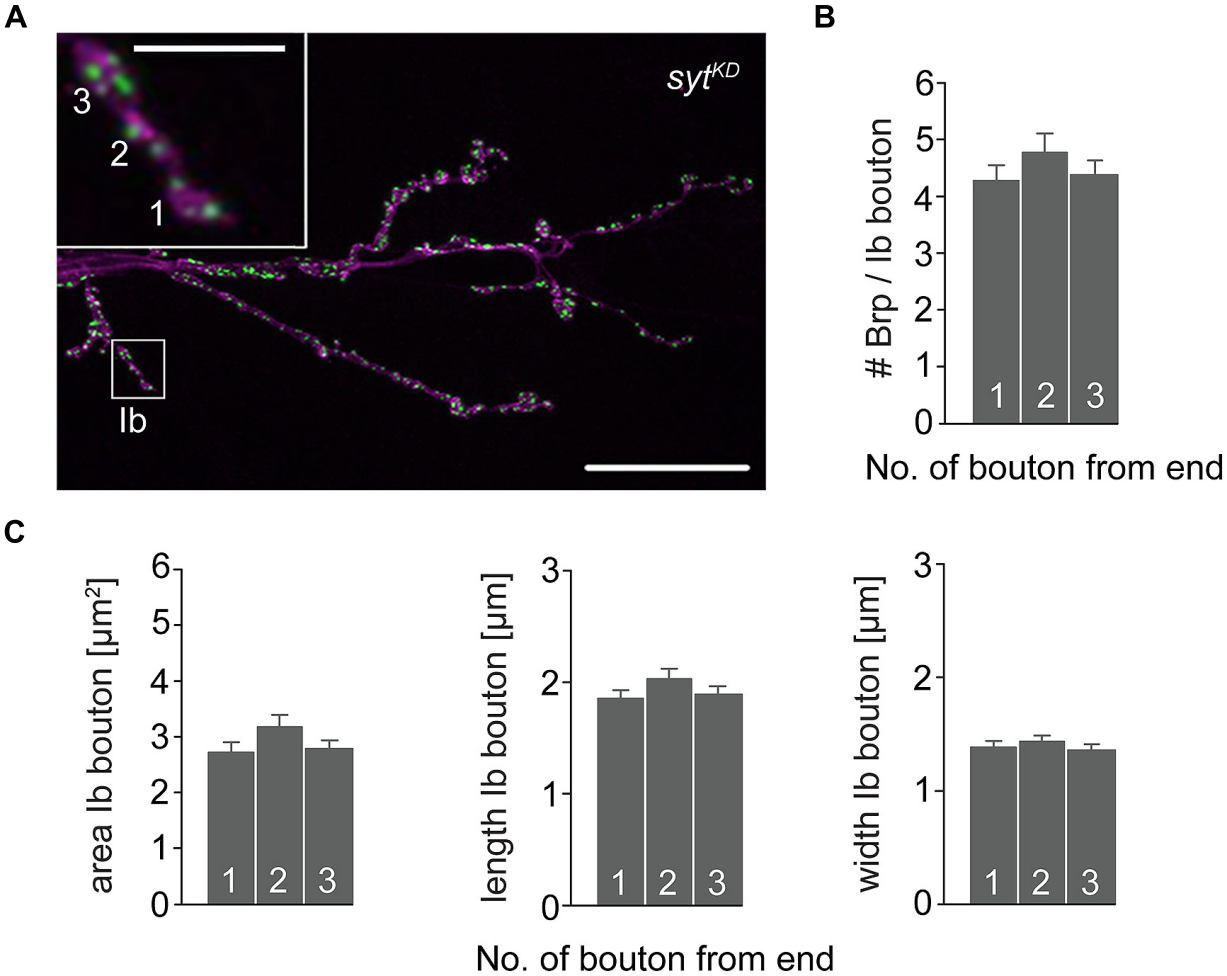
**Type Ib bouton morphometry in *syt^**KD**^*. (A)** *syt^KD^* NMJ stained with α-HRP against neuronal membranes (magenta) and mAb Brp^Nc82^ (green). Higher magnification of type Ib ending (white box) showing the three terminal boutons. Scale bars = 20 μm, 2 μm (inset). **(B)** Summary bar graph for the number of Brp puncta per terminal three type Ib boutons in *syt^KD^* and **(C)** bouton area, length, and width.

To further investigate the impact of Brp and Syt on maintaining the structural gradient we again performed immunostainings (**Figure 8**). At *brp^69^* NMJs, bouton area, length, and width were larger for distal than for proximal type Ib boutons (data not shown). Analysis of *syt^KD^* NMJs showed profound alterations of synaptic morphology regarding Brp distribution and bouton size. Whereas the number of Brp positive AZs per NMJ was slightly decreased compared to wt (724 ± 38 and 861 ± 41, *p* = 0.019, *n* = 16 NMJs each), AZ numbers per Ib bouton were reduced to about a quarter. Furthermore, Brp was distributed homogeneously along the MN6/7b-Ib motor neuron and spatial dimensions of type Ib boutons were similar for all locations along the motoneuron [*p* > 0.05, *n* = 125 (1), 109 (2), 109 (3), **Figures 8B,C**]. Compared to wt, bouton size was reduced dramatically. In addition, analysis of the structural gradient in combined *rab3^rup^, syt^KD^* animals revealed that Syt knock-down also decreases the structural differentation at *rab3^rup^* NMJs (data not shown). These data demonstrate that Syt is essential for the structural differentation of the NMJ both in wt and *rab3^rup^*.

### SYT INFLUENCES ORGANIZATION AND NUMBER OF BRP PROTEINS AT INDIVIDUAL AZs

**FIGURE 9:**
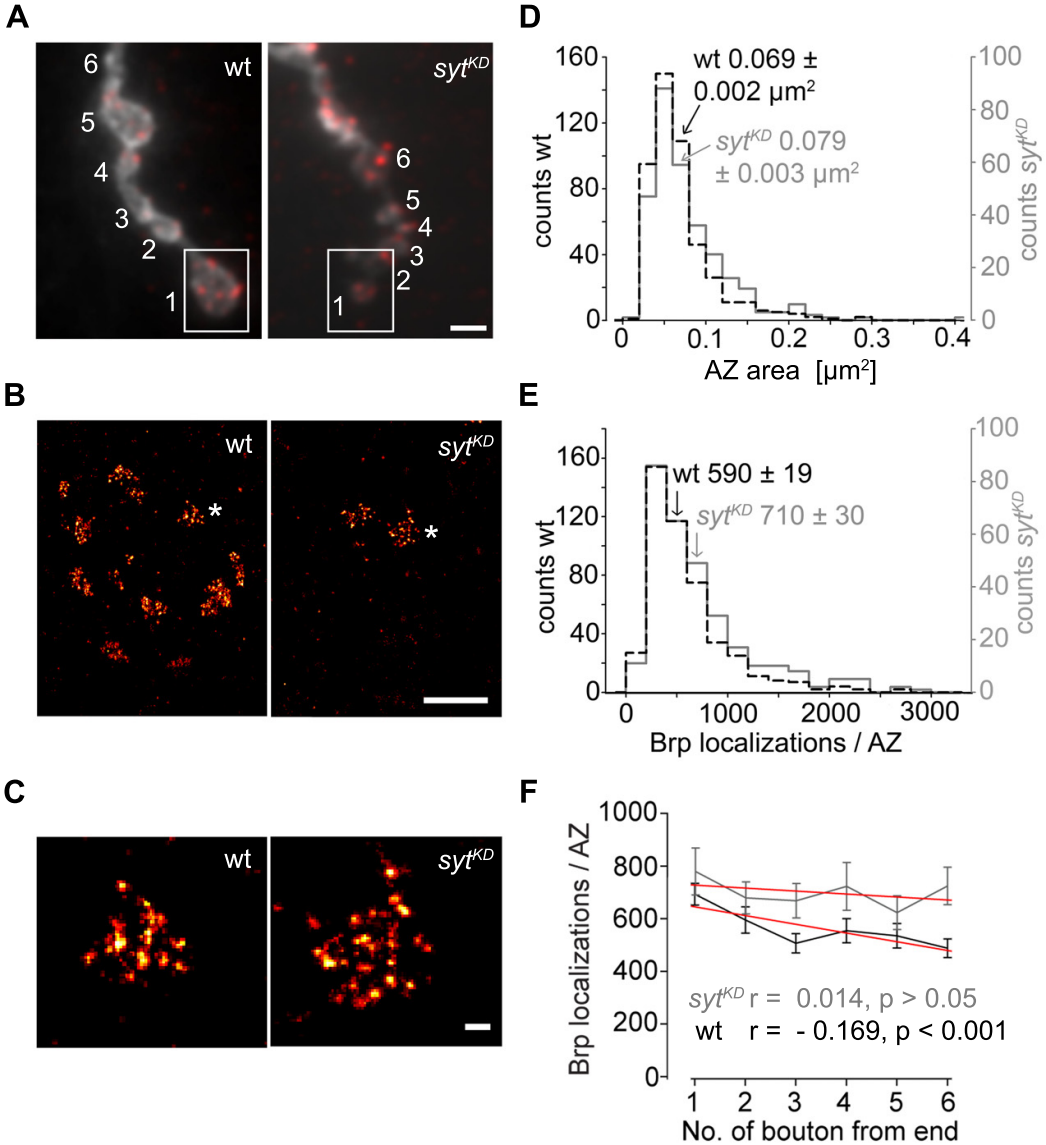
**Super-resolution imaging of* syt^**KD**^* AZs. (A)** Two-channel epifluorescence image displaying the terminal six type Ib boutons of wt and *syt^KD^* NMJs stained with α-HRP (gray) and mAb Brp^Nc82^ (red). **(B)** *d*STORM images of distal boutons (1, boxed region) show individual AZs defined by Brp. **(C)** Magnification of *d*STORM images (asterisks in **B**) show that single AZs are composed of clustered Brp localizations. **(D)** Compared to wt (black), AZ areas and **(E)** distribution of Brp localizations per AZ are shifted to larger values in *syt^KD^* (gray). Arrows indicate respective mean ± SEM values. **(F)** Linear fits (red) of mean ± SEM Brp localizations per AZ in the terminal six type Ib boutons had slopes of –33.2 (wt) and –11.2 (*syt^KD^*) (non-parametric Spearman correlation coefficient wt: *r* = –0.169, *p* < 0.001; *syt^KD^*: *r* = 0.014, *p* > 0.05). Scale bars = 2 μm **(A)**, 1 μm **(B)**, 100 nm **(C)**.

In a final set of experiments we employed *d*STORM to image glutamatergic boutons. This localization microscopy technique substantially increases spatial resolution compared to conventional fluorescence light microscopy (Heilemann et al., 2008; van de Linde et al., 2011) and can provide quantitative insight into the nanoscopic organization of presynaptic AZs (Sauer, 2013; Ehmann et al., 2014, 2015). *d*STORM resolved the substructural arrangement of indiviudal Brp localizations into multiple clusters within single AZs, which correspond to diffraction-limited Brp puncta in confocal images. We analyzed AZs in the distal six type Ib boutons of MN6/7b-Ib motor neurons in wt and *syt^KD^* (**Figure 9**). Interestingly, *syt^KD^* AZs were larger than their wt counterparts (0.079 ± 0.003 μm^2^ and 0.069 ± 0.002 μm^2^, *p* = 0.003, *n* = 300, and 468 AZs, **Figure 9D**) and contained more localizations (710 ± 30 and 590 ± 19, *p* = 0.003, **Figure 9E**), which reflects an increased number of Brp protein copies (Ehmann et al., 2014). Furthermore, we observed that *syt^KD^* AZs contained a similar number of Brp localizations irrespective of bouton order (**Figure 9F**, Spearman correlation coefficient *r* = -0.169, *p* < 0.001 for wt and *r* = 0.014, *p* > 0.05 for *syt^KD^* indicates a moderate negative correlation for wt and no correlation for *syt^KD^*). Thus, Syt influences the arrangement of Brp at the AZ. Previous work showed that the number of Brp localizations per wt AZ is higher in distal than in proximal type Ib boutons (Ehmann et al., 2014). This is consistent with the electrophysiological and structural data presented here.

## DISCUSSION

While differentiation of presynaptic terminals was initially described more than 50 years ago and has since been studied extensively in various organisms, its mechanisms remain poorly understood (Katz, 1936; Hoyle and Wiersma, 1958; Reyes et al., 1998; reviewed in Atwood and Karunanithi, 2002). In view of the enormous complexity of the relevant molecular mechanisms (Südhof, 2012), the genetically and experimentally accessible NMJ of *Drosophila melanogaster* provides advantageous features for studying a glutamatergic synaptic system. Type Is- and Ib-boutons of the NMJ exhibit distinct functional properties (Kurdyak et al., 1994; Pawlu et al., 2004), show differences in vesicle size (Karunanithi et al., 2002) and in the amount of Brp molecules per AZ (Ehmann et al., 2014).

Here we show branch-specific differentiation in the MN6/7b-Ib motoneuron regarding structure and function. Distal type Ib boutons are larger than proximal ones, have more Brp positive AZs and show larger and faster postsynaptic responses (**Figures 5** and **6**). Consistent with these findings, AZs of distal type Ib boutons are larger and possess more Brp molecules per AZ (Ehmann et al., 2014). Presynaptic differentiation is impaired by disrupting either Brp or Syt function (**Figures 6**, **8**, and **9**). Postsynaptic responses of proximal and distal boutons in *brp^69^* and *syt^KD^* are comparable and the structural gradient in bouton size, AZs per bouton and AZ size is absent in *syt^KD^*. Moreover, genetic evidence suggests that Brp and Syt act in the same functional pathway to mediate structural heterogeneity (**Figure 7**). Structural and functional presynaptic differentiation thus clearly requires the concerted action of Brp and Syt.

Interestingly, we obtained consistently lower values for AZ size and Brp counts per AZ than a recent previous investigation using *d*STORM (Ehmann et al., 2014). In the present study, we raised both mutant and control animals at 29°C to ensure efficient RNA-mediated *syt^KD^* (expression via the *GAL4-UAS* system is temperature-dependent), whereas Ehmann et al. (2014) raised larvae at 25°C. Since higher temperature accelerates the development of *Drosophila*, enlarges presynaptic arborizations and increases the number of AZs per NMJ (Ashburner, 1989; Sigrist et al., 2003), it is conceivable that temperature-dependent plasticity also affects molecular organization at the level of individual AZs.

The molecular mechanisms controlling size, structure and distribution of AZs are complex. Several years ago, the vesicle protein Rab3 was identified as an important regulatory factor of AZ size and distribution (Graf et al., 2009). Rab-proteins are key organizers of vesicle trafficking (Harris and Littleton, 2011). Measurements with genetically encoded postsynaptic calcium sensor showed comparable calcium signals in proximal and distal type Ib boutons at *rab3^rup^* NMJs (Peled and Isacoff, 2011). Here, we found a gradient in *rab3^rup^* animals in the number of Brp positive AZs along the MN6/7b-Ib motoneuron (**Figure 5**), unlike in *syt^KD^* (**Figure 8**). Whereas at *rab3^rup^* NMJs the overall number of AZs is reduced dramatically (Graf et al., 2009), this reduction is moderate at *syt^KD^* NMJs (724 ± 38 and 861 ± 41, see Results). However, at both *rab3^rup^* and *syt^KD^* type Ib branches, the number of Brp proteins per AZ is increased strongly and moderately, respectively (Ehmann et al., 2014; **Figure 9**). In contrast to *rab3^rup^* synapses, *syt^KD^* decreases bouton size. Smaller boutons were reported for *syt^AD4^* and linked to defects in endocytosis (Dickman et al., 2006). We found that *brp* and *syt* interact genetically regarding AZ number per bouton, but not area of boutons (**Figure 7**), which suggests that the effects of Syt on bouton size and AZ-differentiation are not strictly linked.

The pronounced presynaptic structural alterations after *syt^KD^* are puzzling. Syt is one of the best-studied synaptic proteins. However, its function has mainly been discussed without considering AZ-differentiation. After *syt^KD^*, evoked release was reduced by a factor of 4–5 compared to wt in our focal recordings (**Figure 6**). However, the number of Brp spots in terminal boutons was also reduced by a factor of 4–5 after *syt^KD^* (**Figure 8**). Is release probability per AZ in distal boutons following *syt^KD^* therefore similar to wt? More work perhaps combining optical release sensors, focal recordings and subsequent immunostainings will be necessary to clarify this issue. The present study highlights how interpretations of synaptic function and differentiation profit from electrophysiological recording techniques with improved spatial resolution (focal vs. TEVC). Functional sampling of synaptic subsets appears absolutely necessary when considering the significant differentiation at the structural level. Either way, linking structure and function at one and the same AZ is fundamentally important for a comprehensive mechanistic interpretation (Bailey and Chen, 1983; Wojtowicz et al., 1994).

Our electrophysiological data suggest that Syt protracts release at AZs lacking Brp. In this study, we used *syt^KD^* to reduce the protein level in presynpatic terminals (**Figure 2**). While we assume there are normally more than 10 Syt molecules on each vesicle in our preparation (Takamori et al., 2006), it is unclear whether *syt^KD^* leads to a reduction in the average number of Syt proteins per vesicle or a reduction in the number of Syt positive vesicles with those remaining possessing a full complement of Syt copies. This is relevant in the context of the molecular interpretation of our results. For example, ring-like oligomerization of Syt’s cytosolic C2-domains, which prevents release in the absence of calcium, requires a certain copy number (Wang et al., 2014). Furthermore, quantitative information on Syt’s partner molecules, such as Complexin and SNARE-proteins, will be required for a mechanistic interpretation down to the level of stochiometric interactions (Mohrmann et al., 2010; Cho et al., 2014). Imaging techniques such as *d*STORM can be used to quantify the molecular organization of AZs (Sauer, 2013; Ehmann et al., 2015) and will be necessary to clarify the so far insufficiently understood kinetic release parameters. In this context, interpretations may well have to take into account the existence of alternative sensors (Walter et al., 2011).

Syt contributes to vesicle docking at the AZ, vesicle positioning within the AZ, clamping and triggering release from the AZ (Walter et al., 2011). While specific amino acids of Syt have been put in connection with certain subsets of these features (e.g., Young and Neher, 2009), it remains unclear which specific functional roles and molecular domains of Syt are responsible for interactions with Brp and structural synaptic specialization. Intriguingly, both Syt and Rab3 are involved in vesicle trafficking and participate in the structural differentiation of AZs. Our work supports the notion that organization of the synaptic vesicle cycle and AZ structure are causally linked.

## AUTHOR CONTRIBUTIONS

Mila M. Paul, Martin Pauli, Nadine Ehmann, Stefan Hallermann, Markus Sauer, and Manfred Heckmann performed experiments. Mila M. Paul, Stefan Hallermann, Robert J. Kittel, and Manfred Heckmann analyzed the data. Robert J. Kittel and Manfred Heckmann conceived the project and coordinated the study. Mila M. Paul, Robert J. Kittel, and Manfred Heckmann wrote the manuscript with assistance from all co-authors.

## Conflict of Interest Statement

The authors declare that the research was conducted in the absence of any commercial or financial relationships that could be construed as a potential conflict of interest.
